# How, when and why abilities go social: researching children’s empathy and prosocial behaviors in context

**DOI:** 10.3389/fpsyg.2023.952786

**Published:** 2023-06-20

**Authors:** Simone Roerig, Floryt van Wesel, Sandra J. T. M. Evers, Anna van der Meulen, Lydia Krabbendam

**Affiliations:** ^1^Faculty of Behavioural and Movement Sciences, Department of Clinical Neuro- and Developmental Psychology, VU Amsterdam, Amsterdam, Netherlands; ^2^Kwalimetrika, Bussum, Netherlands; ^3^Independent Researcher, Amsterdam, Netherlands; ^4^Leiden Institute of Advanced Computer Science, Faculty of Science, Leiden University, Leiden, Netherlands

**Keywords:** children, empathy, context, mixed methods, interdisciplinary research, individual abilities, classroom dynamics

## Abstract

**Introduction:**

The current paper undertakes interdisciplinary research on empathy in children by combining insights and methodological tools from the fields of psychology, education and anthropology. The researchers aim to map how children’s individual empathic abilities studied on a cognitive level do or do not coincide with their empathic expressions as part of group dynamics in daily life at the classroom level.

**Method:**

We combined qualitative and quantitative methods within three different classrooms at three different schools. In total, 77 children aged between 9 to 12 years participated.

**Results:**

The results indicate how such an interdisciplinary approach can provide unique insights. Through the integration of data from our different research tools we could reveal the interplay between different levels. More specifically this meant showing the possible influence of rule-based prosocial behaviors versus empathy based prosocial behaviors, the interplay between community empathic abilities and individual empathic abilities, and the role of peer culture and school culture.

**Discussion:**

These insights can be seen as encouragement toward a research approach that extends beyond the single disciplinary field in social science research.

## Introduction

Since the advent of the social brain concept, brain scientists have become increasingly interested in the social sciences, and this is an opportune time for interdisciplinary collaboration (Whitehead, 2012: 64).

### Research overview

Interdisciplinary collaboration is the starting point of the current study. We aim to integrate theoretical perspectives and methodological approaches from the disciplines of psychology and anthropology to study empathy in children. Human empathy refers to the capacity to understand and/or feel what others feel, from within their frame of reference. It is essential for children’s social and emotional development and also for successfully starting and maintaining social relationships (Eisenberg, 2000). Empathy is a widely debated topic in studies of human interaction in multiple scientific disciplines, including neurosciences, psychology and anthropology. Interestingly, the differences in approaching the study of empathy are considerable. While in neurosciences and psychology empathy is seen as a trait and/or state that in all its varieties belongs to the individual (Cuff et al., 2016; Coll et al., 2017), studies in anthropology consider empathy a process that emerges between interacting individuals. These distinct conceptual views lead to different research aims and methods. Research traditions in neuroscience and psychology aim to disentangle components of empathy, their instantiation in the brain, the developmental trajectories, genetic underpinnings, and the causes and consequences of impairments in components of empathy (Davis, 1983; Eisenberg, 2000; Decety and Jackson, 2004; Singer, 2006; Lieberman, 2007; Blair, 2018; Warrier et al., 2018). In contrast, studies in anthropology investigate the observable elements of expressive empathy between empathizer and empathized in daily life situations and the influence of social and cultural context on this empathic expressions (Astuti, 2007, 2015; Groark, 2008; Hollan and Throop, 2011; Roerig et al., 2015). Both perspectives increase our understanding of different aspects of empathy, yet by investigating them in isolation valuable insights may be missed. Therefore, the current study aimed to offer an exploration of how both perspectives can be integrated. In order to obtain this, we have used methods from psychology and anthropology in the study of empathy.

#### Contribution

Before we introduce our research questions, we will give some context on the different perspectives within the topic of empathy that we will integrate. With the integration of perspectives as mentioned above we hope to contribute primarily to the broader study of empathy by investigating empathy both as an intra-individual ability in an experimental context, and as an observable inter-individual process in a daily life context – in this case, children’s school lives. The idea of combining insights from the psychological aspects of human interaction and cultural and social real-life factors is not new (Benedict, 1938; Batson, 2009; Zahavi 2010; Engelen and Röttger-Rössler, 2012; Hollan, 2012; Roepstorff and Frith, 2012; Gilin et al., 2013; Roerig et al., 2015; Winczewski et al., 2016; Bethlehem et al., 2017; Scheler, 2017). However, most of this (interdisciplinary) work on empathy is based on experimental measurements and staged behavioral settings as opposed to empathy as it is observed in people’s daily lives (Hollan and Throop, 2008; Bethlehem et al., 2017) which, resulted in overlooking empathy as an interpersonal, dynamic and relational construct (Main et al., 2017). In our study we therefore want to commit to ‘find(ing) creative and ecologically valid ways to deepen our understanding of this important topic’ (Main et al., 2017, p. 364). We do this by employing both the unit of analysis that is common for psychologists – the abilities of the individual child – and the unit of analysis that is common for anthropologists – the community or culture, in our case the children’s classroom (Haneda, 2006). The classroom will be treated as a community of practice (Lave and Wenger, 1991; Haneda, 2006) as related to the situated learning perspective (see among others Lave and Wenger, 1991) in which individual cognitive ability and social practice are said to co-create each other. Understanding and expressing empathy then is not just an individual ability but part of a collective classroom ability. By researching, analyzing and discussing both these abilities in one paper we believe this is a unique chance for anthropologists, psychologists and educators to unite.

In the current study, we investigate the inter-individual nature of empathy with qualitative and quantitative approaches. We make use of qualitative (observational field notes) and quantitative (social cognition tests) measurements, but we also add the use of social network analysis (SNA) to quantify the qualitative relationships between children. SNA combines qualitative and quantitative starting points since it focuses on both relationships and computed patterns (Marin and Wellman, 2011). Whereas more one-dimensional approaches focuses on for example characteristics of the relevant actors within each organization (e.g., “this child is always very shy”), SNA focuses on the diverse connections between the actors of the various organizations leading to new insights. Social networks are formed by nodes (network members) that are tied by relations (Wasserman and Faust, 1994). The nodes are most commonly persons or organizations, but can in principle be any other unit (articles, countries, positions). Relations can be any possible connection between the nodes, but Borgatti et al. (2009) distinguish a useful four categories of relations: similarities (e.g., shared characteristics), social relations (e.g., friends, liking or knowing), interactions (e.g., speaking, helping) and flows (e.g., exchange of any sort). In our study we investigate two categories of relations namely social relations and interactions. The use of SNA can help us to further understand peer interactions in relation to empathy (Meyer et al., 2013). In educational research peer relationships are often indicated through peer nominations (Martín-Antón et al., 2016) and sociograms (Sobieski and Dell'Angelo, 2016) and the SNA approach ties in with this kind of research. SNA embodies interdisciplinarity by combining nodes (individual level information), links (relational information), and graphs (group level information) (Fotopoulou et al., 2021b, p. 17, p. 23). The rich information of social networks and especially children’s peer relations within a classroom is often related to interventional and educational use. For example, classroom climate and children’s peer interactions are said to affect children’s school learning (Leung and Silberling, 2006), a link that was already researched decades ago by Moreno (1953). Also, social network knowledge as examined through SNA can be directly linked to empathy related aims, for example, to enhance social skills, increase empathy or as a signal to educators for possible aggressive or bullying behaviors (Fotopoulou et al., 2021b, p. 3, p. 5). And, the position of children within an interactive social network – such as a classroom – has been associated with cognitive and affective empathy (Wölfer et al., 2012). In the current study we similarly link SNA to empathic measures, but also relate this information to observational information on children’s empathic behaviors to innovatively gain insight in the link between intrapersonal and interpersonal empathy in the context of a group. Together with Fotopoulou et al. (2019) and Fotopoulou et al. (2021b) we believe the direct embedding of an individual empathic profile of a child in the context of classroom dynamics can add to the existing literature and scientific practice for psychologists, but also for anthropologists and educators.

Finally, we investigate empathy in a relatively understudied developmental population, namely children from 9 to 12 years old. In this age range children are generally capable of understanding complex social emotions, are aware of the needs of others, able to show empathic behavior, and capable of the basic reasoning associated with prosocial behavior (Decety, 2010). At the same time they show ongoing development of more advanced forms of social reasoning such as perspective-taking (Harris, 1994). Moreover, children in this age group have a certain social awareness and can reflect on the social rules they implicitly or explicitly use, and relationships with peers are paramount in their lives (Hay et al., 2004; Banerjee et al., 2011). For example, Demetriou (2018), comparing 7, 8 year olds with 9, 10 year olds, explains how in comparison to the younger children who do recognize and understand emotions, the older children understand more about the complexity of emotions and are more sensitive to moral and social codes related to empathic behaviors (Demetriou, 2018:98). Furthermore, children of this age are capable to undertake social cognition tests and questionnaires (Banerjee et al., 2011). This allows using various psychological paradigms to assess empathy, including self-report, in a reliable and valid way (Bajgar et al., 2005; Castro et al., 2016; Van der Meulen et al., 2017).

In sum, the aim of this study was to investigate empathy as an intra and inter individual construct in middle childhood, using ability tests and questionnaires, observational methods and social network analysis. Embedded within this background, which will be further elaborated on in the theoretical framework section, two research questions form the core of the study:

1. How is the child’s individual ability to empathize related to their empathic expressions as observed in the socio-cultural environment of their daily school lives?
2. How can the use of social network analysis help us to understand the interplay between empathy as an individual construct and empathy as part of a classroom community of practice?

In the next paragraph we elaborate more on the theoretical underpinnings of this study, discussing the different approaches to empathy across fields. Next, we present the details of the methodological approach, followed by the results and discussion section and our concluding remarks.

### Theoretical framework

#### Empathy as an individual ability: strengths and weaknesses

Decety and Lamm (2006: 1146) describe empathy as an individual ability ‘to experience and understand what others feel without confusion between oneself and others. In the work of psychologists, a key distinction is made between cognitive aspects of empathy (i.e., reflecting on the emotional state of others) and affective aspects of empathy (sharing the emotional state of the other) (Ahn and Goh, 2010). Affective empathy is often further divided in personal distress (see IRI, Davis, 1983) or a self-focused negative state in response to the emotional state of the other, and empathic concern (see IRI, Davis, 1983 Jordan et al., 2016) an other-focused emotional response that is distinct from the emotional state of the other. Neuroscientific research has suggested that these different components have different neural substrates (Zaki and Ochsner, 2012). Different components of empathy may also be differentially related to prosocial behaviors (Jordan et al., 2016). A key assumption in psychological work on empathy is that the various aspects of empathy can be considered as a rather stable part of an individual (Ramaswamy and Bergin, 2009). Convincing work on empathic abilities suggests that there are indeed trait individual differences in empathy (Cuff et al., 2016). In combination with insights into how this trait can develop over time, we learn that there is a baseline basic ability (trait), which is a precondition for the individual to be actually able to express empathy in a particular situation (state). An important aim of this individual ability approach is to obtain a good estimate of these abilities, which can then be linked to other individual traits, states or behaviors (Yang et al., 2009; Marsh et al., 2013; Van Doesum et al., 2013; Keysers and Gazzola, 2014; Winczewski et al., 2016; Bethlehem et al., 2017; Coll et al., 2017). Usually the tools to obtain such a good estimate of empathic abilities are standardized self-reports and/or experimental tasks. Both take place in controlled settings that are either devoid of contextual influences or controlled by manipulated situational influences. Main strengths of the empathy as individual ability approach are: (1) behavior can be linked to diverse empathic components and abilities that can be studied using experimental approaches and (2) the possibility to study individual differences for example related to developmental phase and individual characteristics such as sex or gender (Yang et al., 2009) and forms of psychopathology (Marsh et al., 2013). It can be noted that within the discipline of psychology itself there is not necessarily always clear consensus on the construct and measurement of empathy (Blair, 2005; Prevost et al., 2014). The most important weakness of the approach is the focus on empathy *within* the individual, whereas empathy is said to be both an intrapersonal and an interpersonal construct. The latter approach – in which empathy is construed as a process – is much more highlighted in anthropological work.

#### Empathy as an interactive process: strengths and weaknesses

Hollan and Throop (2008) describe empathy as ‘an ongoing, dialogical, inter-subjective accomplishment that depends very much on what others are willing or able to let us understand about them’ (Hollan and Throop, 2008: 394). In other words: empathy is an interpersonal process. The main focus in anthropological research is to understand how these processes may then inhibit or enable expression of empathy in a given situation (Hollan and Throop, 2011). Hereby the empathizer and the person empathized with are equally valued (Groark, 2008) and placed in a context of socio-cultural processes. In the fieldwork of Throop (2010) on the island of Yap, the author brings in his own grieving experiences to imply how empathy is “a process that is temporally arrayed, intersubjectively constituted, and culturally patterned” (Throop, 2010:771). He argues that one’s lived realities influence one’s abilities to empathize at all times and advocates the perspective that empathy can seldomly be seen as “an all or nothing affair” (*ibid*). Anthropologist Briggs (2008) explains how, among the Inuit, publicly expressing one’s emotions is seen as something childish. An adult might thus be able and even willing to express his or her feelings but might not be allowed to do so in a particular public context involving particular people from his or her social network. In their recent interdisciplinary work on the socialization of emotions, Röttger-Rössler et al. (2015) describe the process of ‘who may feel which emotions when, with which intensity, and in front of whom they should be expressed’ as ‘feeling rules’. It is within the variation of these ‘rules’ that the anthropologist gains greater insights into people’s expressed empathy and the function of it in their everyday community practices (Main et al., 2017). The strength of the anthropological approach is that empathy is researched within the context of daily life dynamics and everyday practices: interactive processes are the core of investigation. While ‘daily life’ components of the individual in psychological studies are often measured through experimental methods, questionnaires and/or stories using *fictive* others or actors, anthropologists attempt to gain access to the ‘daily life’ of *real* others, thereby investigating expressed empathy. The weakness of this approach is that the individual ability to empathize and the developmental trajectories of children’s empathy are often not incorporated, so individual (dis)abilities, boundaries or challenges can be overlooked or wrongly be interpreted as part of an interpersonal process.

#### Empathy in the classroom: children are agents, community of practice and empathy as a function

In our research we want to capitalize on the strengths of both the psychological and the anthropological approaches. The context of the research is the classroom and we believe this is an ideal place theoretically and practically to execute our research. First, the focus on individual and interpersonal and their interaction fits naturally within an educational context (c.f. Haneda, 2006). Expressing and understanding/knowing empathy is not just an individual ability, but a part of the community ability of a classroom. Second, the classroom is the setting where children spend most of their social life and thus a natural habitat for the study of expressing empathy and using a social network approach. Third, the classroom can be seen as a community of practice (Lave and Wenger, 1991; Haneda, 2006). In the context of describing the cultural-historical approach toward the study of learning, Gutiérrez and Rogoff (2003, p. 23) explain how indeed the link between community’s practices and everyday practices an individual undertakes is paramount. Children learn how empathy can best be used in the context of their classroom and thus the way children express empathy can have a certain aim within the classroom dynamics. Hereby, learning empathy and the way in which empathy is appropriately expressed within the context of the particular classroom becomes part of a social routine (Gutiérrez and Rogoff, 2003:23) and the community norms (Payne et al., 2020:36) of a classroom. In our research toward children’s classroom beliefs, positions and behaviors we want to emphasize that we see children as citizens who are in general able to care for each other and their communities in meaningful, non-adult led ways that children create and execute (Payne et al., 2020:38).

#### Empathy in context: bringing together abilities, processes, intra- and interpersonal empathy

Hollan (2012) already considered the call to combine the dynamic inter-relationship between trait and expressed empathy within one study to be highly relevant. Others followed (see among others Winczewski et al., 2016 Bethlehem et al., 2017 Main et al., 2017). Conceptualizing empathy as an individual ability or as a mutual effort within an interactive process leads to fundamentally distinct approaches. To capitalize on the strengths of both approaches, we will investigate children’s empathy at their individual ability level while embedded in the context of their classroom lives, where empathic expressions are shaped in the interactive processes with their peers. In conjunction with Cuff et al. (2016, p. 150) we believe empathy to be a result of the interaction between state capacities and trait influences and hope to find how knowledge of individual children’s scores on decontextualized, lab-based studies of their abilities to feel empathy can add to our understanding of classroom dynamics and vice versa how classroom dynamics can influence, match or mismatch with individual ability empathy scores. Together with Lave and Wenger (1999:33) we see how the interplay of our three levels of investigation is crucial since “agent, activity, and the world mutually constitute each other.” In the following section, we will elaborate on the research design we developed to pursue the exploration of our interdisciplinary research quest.

## Materials and methods

### Design

Building on an interdisciplinary design, by which we aimed to investigate empathy on different levels using research techniques from different scientific fields, the use of a mixed methods approach (Johnson et al., 2007) was most suitable for our study. Morse and Niehaus (2009) define such an approach as a study wherein multiple methods are used because the use of a mono-method does not suffice or is not exhaustive (2009: 9). The type of mixed methods design we use is a fully nested sample design, meaning that we have all types of data for all our participants, in which the weight of quantitative and qualitative components is equally valued (QUAN+QUAL) (see for notation Creswell et al., 2003). The investigation of individual empathic abilities or skills of children, as discussed above, is based on existing psychological tasks which are mainly quantitative in nature. The empathic interactions were assessed *via* non-structured participant observation.

### Sample

This research took place at three different schools in the Netherlands. Part of the collected data – the LEAS(C), social network analysis and observational data from one classroom – was included in a short paper on the value of a mixed-methods approach in empathy research (Roerig et al., 2015). In the 2015 paper the focus was on individual children and the analysis was based on a within-classroom comparison. The current data set encompasses three classrooms and focuses on a between-classroom comparison. Due to this focus the description of classroom dynamics and the presentation of qualitative data play a far more important role in the current paper. We purposefully included schools from different geographical areas. The three classrooms we worked with were recruited *via* the first author – who is also a middle school teacher. Within her network we selected three schools with sufficient variation in location in terms of population density, and social and economic neighborhood characteristics. Further, it was important that principals, parents and children wanted to participate. The first author explained the research to the children in the classrooms before starting the research. Children were able to ask questions about the participation (which they did) and chose *via* a consent-paper if they wanted to participate themselves. School 1, Classroom 1 (Cl.1) (15 girls, 13 boys) was in a smaller village close to a big city. Income in the school area was between 33.500 and 35.800 which is above Dutch average (26.000) and citizens in neighborhood were from 0.8% non-Western and 99.2% Western background. School 2, Classroom 2 (Cl.2) (14 girls, 13 boys)was situated in a more rural, not too small, town. Income in the school area was between 30.200 and 30.500, which is lower than in Classroom 1, but still above Dutch average. In this neighborhood 2% of the citizens had a non-Western background and 98% had a Western background. School 3, Classroom 3 (Cl.3) (16 girls, 6 boys) was in an urban environment. Income in the school area was between 23.800 and 25.600 which is below Dutch average. In the neighborhood 28–44% had a non-Western background, whereas 56–72% had a Western background. In total our sample of three classrooms consisted of 77 children, which can be considered a sufficient relatively large sample for qualitative research (Vasileiou et al., 2018). For the quantitative part of our study, we adjusted our analysis approach to the sample size as described below.

### Materials

We used three measurement instruments:

1. Reading the Mind in the Eyes test for children (RME-C, Baron-Cohen et al., 2001), investigating children’s ability to recognize and define the mental and emotional states of others based on facial cues (Van der Meulen et al., 2017). In the RME-version we used, 14 pictures of eyes were shown to the participant. Each picture depicts a certain mental state/emotion and each of these picture is escorted by four words that represent a possible mental state. Participants choose one of these four mental stated that they think is most in line with what they see in the picture. When the answer matches the depicted state, the child scores 1 point, the range is 0–14 points. RME (Child) has several reported Cronbach alpha’s (Van der Meulen et al., 2017), but not in the original paper (Baron-Cohen et al., 2001). In the current sample Cronbach’s alpha of the RME (Child) was 0.383 (14 items, *N* = 77).
2. Level of Emotional Awareness Scale for children (LEAS-C, Bajgar et al., 2005) to measure their ability to understand the complexity of emotions, by distinguishing between feelings of self and others (on the cognitive level). LEAS-C includes 12 scenarios on which children report their feelings about themselves and the other person in the story. No response or a description of cognitions equals 0 points, description of a bodily sensation equals 1 point, description of a global hedonic state equals 2 points, description of an unidimensional emotion equals 3 points, description of differentiated emotions equals 4 points and description of more complex and differentiated states equals 5 points (Bajgar et al., 2005:577). The range is 0–60 points. According to Bajgar et al. (2005), Cronbach’s alpha was a 0.71 for self-scores, a 0.64 for other-scores, and 0.66 for total scores (*N* = 51)” (Bajgar et al., 2005:579). In our sample, Cronbach’s Alpha was 0.81 for self-scores, 0.80 for other-scores and 0.83 for total scores (12 items, *N* = 77).
3. How I Feel in Different Situations (HIFDS, Caravita et al., 2009): a self-report scale on children’s affective (5 items) and cognitive (6 items) empathic abilities. Participants get to see a statement such as ‘seeing a friend crying makes me feel as if I am crying too’ (*ibid.*) and then decide how much this statement would be applicable to them: from 1 = never true to 4 = always true. The range is 0–44 points. In Caravita et al. (2009) which we derived the task from (from the appendix of that paper) no Cronbach’s Alpha is reported. Based on the current sample, Cronbach’s alpha was 0.784 (11 items, *N* = 77). To study children’s empathic expressions in their everyday context, we used social network analysis (SNA) in combination with classroom observations. By the use of SNA we investigated the social context in the classroom in a quantitative way (Fotopoulou et al., 2021a). All participants received a list of names of children in their classroom and for every child reported reported either a like, a dislike, or a neutral relationship for each of their classmates. Moreover, they recorded to whom of their classmates they would go to if they needed help.

In addition to the SNA we used qualitative field notes that were written during the classroom observations. In observing children’s empathic expressions during their daily interactions, we focused on their immediate context – what happens during the school day – rather than the long-term context – the set of norms and values they are taught by those who raise them. The observations were open and unstructured in a way that the researcher noted all behavior that was related to the above empathic and prosocial elements and the coding only took place after the observations. Observation included all day activities in and outside the classroom, such as lunch breaks, playing in the school yard and physical education lessons. The observer sat in the classroom on different places, sometimes participated in classroom activities and had informal talks with the children on a regular basis. Although the observer participated in some activities with the children, the observations are not comparable to an ethnography in which the researchers becomes part of the classroom life. The focus of the observations was to record the children’s prosocial behaviors, empathic expressions and classroom dynamics. In this research prosocial behavior is understood as “(…) any behavior that benefits others, such as sharing, cooperating, including others in play, complimenting, and comforting others” (Ramaswamy and Bergin, 2009, p. 527). Moreover the focus was on mapping the classroom as a community of practice including which empathic rules are important in the classroom or which social interactive behavior belongs more or less to the group identity. On the cognitive level we expect to see the empathic expressions concern and comfort that are in this paper considered to be related to perspective taking (Jordan et al., 2016). On the affective level we count sharing emotions: crying with/for others, laughing together, being angry with others, as empathic expressions.

### Procedure

For all schools and classrooms, we required written informed consent from both the children and their parents. We individually asked every child and parent for consent (Cl.1/2 active, Cl.3 passive) and in Cl.1 and Cl.2 participation rate was 100%, in Cl.3 92%. The parents of two children did not want their children to participate. The study was approved by the Ethical Committee of the Faculty of Behavioral and Movement Sciences of the Vrije Universiteit, Amsterdam. The three psychological tasks and the social network task were carried out in a random order by each child individually, outside the classroom, with only the researcher present and on the basis of complete confidentiality. The quantitative measures were administered by the first author together with a colleague (Msc.) who was also trained in social science (psychology/pedagogy). Per classroom all children completed these tasks on the same day and in total, it took participants 30–45 min to complete the tasks. The observations were conducted by the first author (who is trained in social science and working with children) who was participating in the classroom for 1 day a week for a period of 3 months.

### Data analytical approach

In our analysis, we established the individual high and low scorers on the psychological tasks for the whole sample by computing z-scores with the Statistical Package for the Social Sciences (SPSS), version 20. Based on the means and sum scores of the three cognitive tests – LEAS-C (Cronbach’s Alpha 0.81 Self, 0.80 Other, 0.83 Total), RME (Cronbach’s Alpha 0.38 – this task is known for this problem see Van der Meulen et al., 2017) and HIFDS (Cronbach’s Alpha 0.78) – children’s individual abilities were analysed and mapped by computing z-scores for the three classrooms as a whole. For every ability task, we listed the individuals whose standard deviations were above or below 1. The children who scored above or below 1 on *all* three psychological tasks (RME, LEAS-C and HIFDS) were labelled high or low scorers, respectively. To perform the social network analysis, we used UCINET (Borgatti et al., 2002) and NETDRAW (Borgatti, 2002). In the network analysis, we calculated indegree and outdegree of likes, dislikes and help questions, where indegree equals the number of children who like/dislike the participant or asks the participant for help, and outdegree equals who the participant likes/dislikes or asks for help. We used one-way ANOVAs to examine social network differences between classrooms (*n* = 28, *n* = 27, *n* = 22). The qualitative field notes collected through observation were thematically analysed using open, axial and selective coding (Corbin and Strauss, 2008). We worked with two coders (one with an anthropological background and one with a psychology/methodology background) and made use of so called researcher triangulation. To arrive at a specific code the coders go through three steps. First, one reads the field notes on a general level and links certain text-pieces to a code. For example in Cl.1 there is a field note about girls who not let everyone in at their play, the code is: ‘exclusion’. Second, when a code like ‘exclusion’ (or ‘inclusion’) repeatedly comes up, the coders look at the notes to find all the examples of in-and exclusion and look for anecdotes that match this code. Third, the coders come up with a code name that covers all observations and can be related to the broader theme of the study toward empathy. In this case this overarching code name would be: in- and out-group. Since the coders have an overview of how often this happens in the three classrooms and of the anecdotes the code and its meaning in the text can now be easily inserted.

## Results

Below, we will first present the observational data, which is subdivided into three sections: (1) Shared practices: classroom identity; (2) empathic practices: classroom processes; (3) individual practices: classroom cases. Second, we present children’s individual ability scores and third we present the social network analysis per classroom. Both ability scores and social network outcomes will be discussed in relation to the earlier described observational findings.

### Observations 1. Shared practices: classroom identity

Guided by the work of Payne et al. (2020) in this subsection we try to concretely describe which community norms (Payne et al., 2020:36 – the example of Ms. Luz and Angelica) and shared practices are most important in each classroom. We explicate these norms in three keywords per classroom. These words form an important analytical tool in pinpointing the classroom identity in relationship to the empathic processes and interactions per classroom.

#### Classroom 1

In Classroom 1 (Cl.1), it was important to give the right answer – “I know, I know I know” –, whether it was a mathematics-related question or a social behavior-related question. Children tended to competitively discuss their answers with each other and the teacher. One day during a change-over between two subjects – which requires changing places, using other books, etc. – was taking too much time and the teacher asked the class whether and how they could speed up the process next time. Different ideas were brought up by the children and eventually the ‘kudos system’ (Dutch: ‘pluimensysteem’) was chosen: the first subgroup (the class was divided into 7 subgroups) to be ready for the next subject would receive a ‘kudo’. Once a group collected five ‘kudos’ they would be rewarded, being allowed to choose a 15-min activity that they like. During the first subsequent change-over, the competitive drive of the children immediately guided their behavior: everyone within the subgroup helped each other to prepare as fast as possible and when the fastest group received a ‘kudo’ they celebrated loudly: “Yeahhh we are the champions!!.” Interestingly, the children came up with the ‘kudos system’ themselves, which implies that they knew how important being the best/fastest/highest was for them. Three keywords for Cl.1: competition, punishment/reward, performance.

#### Classroom 2

In Classroom 2 (Cl.2), the children invited the researcher to join in their play, made compliments about the researcher’s clothes – “Miss, I really like your shirt” – as they would do to their peers – “Wow I love your new hair cut” –, and were quite open in sharing their thoughts and feelings. The enthusiasm with which they approached the researcher as an adult and the character of their games sometimes gave the impression that they were younger than their peers in Classrooms 1 and 3 (which was not the case). Moreover, the tendency to give compliments rather than to make a sarcastic joke about someone’s clothes (Cl.3) or only give compliments to your friends (Cl.1 and Cl.3) gave a different feel to the classroom dynamics. Additionally, the teacher in Cl.2 had a different position in the classroom interaction compared to the other teachers: he came across as not so much an equal partner in dialog (Cl.1) or a potential power rival (Cl.3), but a good-hearted authority whom they respected, immediately listened to and with whom they shared their weekend stories. Three keywords for Cl.2: being nice, team spirit, enthusiasm/naivety.

#### Classroom 3

Classroom 3 (Cl.3) field note:

If I were to describe this classroom, I would say it is a snowy landscape. Fickle – you can lose your way or be severely injured. You can become buried in avalanches of words and jokes. But, if the sun comes out, it is the most beautiful place.

In Cl.3 often the peer-to-peer and the child-teacher interactions resembled avalanches: a child or the teacher would say something, someone else would respond, and immediately someone else would react to that response, and so on. During these interactions, power dynamics played an important role: the children who joined in the conversation often attempted to make the funniest, loudest or most shocking remark. This resulted in a certain flow of negative, sarcastic or harsh remarks at the cost of someone in the classroom. Below, is an illustrative conversation between two boys:

•Yassin*^1^: ‘You think you are a king, acting all tough’.

•Marouan: ‘You are such a drama queen’.

•Yassin: ‘Shut up you Moroccan’.

However, the direct and outspoken responses could also be warm and concerned, which occurs, for example, when Issrae did not come back after school holidays, because her mother – without telling anyone – had decided to stay permanently abroad. In the one-on-one conversations between the researcher and both ‘the quiet’ and ‘the loud’, this warmth also predominated. Three keywords for Cl.3: power position/ status, competition, acting tough/two faces.

### Observations 2. Empathic practices: classroom processes

In the description of the classrooms there were three themes that were of significant influence of the empathy-related processes in the classroom: (1) ingroup versus outgroup, (2) adult rules versus child rules, (3) the negative versus the positive. The differences between the classrooms in relation to these three themes seemed crucial for the different community empathic abilities that were observed when we compare the classrooms on empathy-related processes.

#### Ingroup versus outgroup^2^

In Cl.1, there were continuous processes of inclusion and exclusion in the ingroups, which affected empathic behaviors, especially among the girls. This can be exemplified by two situations:

1. During physical education: Inge, who is not so popular, falls and starts crying. She is clearly ignored by all children except for Marianne (her best friend), who pats her on the back.
2. In the changing room (before physical education): Zoë asks Bente: ‘Is Julia still with the blondies?’ There are two groups, the blondes and the brunettes – a subdivision within an ingroup of girls. A short discussion between four girls who are obviously *in* follows, and the answer is ‘Yes, Julia is “in”’. A little later, the same girls are playing football outside. The rule is that only four people can join a team. It turns out that Julia has to leave. She walks away, angry, disappointed and with outrage on her face she shouts: ‘I will never join you again’.

The processes of inclusion and exclusion influenced the way the girls treated each other in certain situations and whether they did or did not express care, comfort or understanding. There were even envelopes with notes about other classmates that circulated among several ingroups, dividing subgroups within larger ingroups. For an outsider, this may appear quite complex, but the girls themselves seemed to know how to adapt their empathic expressions to who was ‘in’ or ‘out’ in that particular context. Generally, boys and girls tended to be in each other’s outgroup. In contrast to the strong and continuous ingroup- and outgroup dynamics in Cl.1, Cl.2 generally operated as one group of individuals. Although friendship ingroups- and outgroups, gender divisions and social exclusion were present, configurations of children who played together changed from day to day and just as often depended on the type of game that was played as on who was playing the game. Again two situations illustrate this finding.

1. In the classroom: All of the children listen attentively to the talk that Yvonne gives on her favorite sport, judo. When Yvonne shows some moves on the judo mat she brought along, she needs a volunteer. Marc spontaneously pitches in to help her. Some boys joke about the girl-boy situation that appears when Marc puts on the judo kit that Yvonne brought along, but Marc does not seem to care and takes his volunteer role very seriously. The rest of the classroom ignores this possible girl-boy ‘thing’ and remains interested and Yvonne is given a high grade and generally receives positive responses from the classroom.
2. At the playground: A mixed group of boys and girls are playing the game ‘kiss for a bliss’. Hiske quits the game because she does not feel like playing anymore and feels some boys are acting childish. She realizes, however, that Christiaan, who is apparently in love with her, might think that she quit because of him. She shares her concerns with me and tells me that she is not in love with him but she also does not want to hurt him. Christiaan stops playing the game as well and indeed looks a bit disappointed. He sits near the sandpit for a while, and Tom comes over to him with a serious face and asks: ‘… What’s the matter? … Are you heartbroken?’ Christiaan denies this and says that he lost something in the sandpit and was looking for it. Tom then leaves it at that and asks Christiaan to join in another game.

In Cl.2 the existence and dynamics of the different in- and out-groups did not constantly define their empathic expressions. Hiske, a non-friend and a different gender, showed empathic concern for Christiaan, as did Tom, a friend of the same gender, while Marc offers his help pro-socially to Yvonne, a non-friend and of a different gender. Generally, creating groups seemed an organic process, in which the children took responsibility not to show antipathy or disappointment when put into the same group as someone who might not belong to their ingroup. In Cl.3, there was some alternation between exclusively showing empathy toward the ingroup – resembling Cl.1 – and showing empathy toward children in the classroom who are outside their particular ingroup – resembling Cl.2. An additional outgroup that is apparent in Cl.3 is the group of teachers and/or adults. Cl.3 does show struggles between children and subgroups are part of the classroom structure, however there are multiple moments during the observations in which the group acts as a whole *against* the adult power or adult rules. In the following two subsections this will be discusses.

#### Adult rules versus child rules

In Cl.2, the tendency was to stick with the social rules that the teacher had set: to have respect for the teacher and your peers, be nice to one another, encourage each other and help each other. Of course, there were exceptions (e.g., when there was another teacher or an individual child had a bad day), but generally the children agreed with the set rules. A different pattern of incorporating the social rules was seen in Cl.1. Although the social rule to ‘Consider other people’s feelings and talk about it if necessary, even when you find this difficult’ was clearly articulated by the teacher and in word agreed upon by the children, they were exhibited far less in acts by the children in the teacher’s absence. The following conversation is illustrative:

- Nynke handed a list of ‘bullies’ to the teacher and the teacher discussed the matter with the classroom.
- Coen: ‘Yes, but it was only meant as a joke … [he thinks before finishing his sentence] but it didn’t feel like a joke to her’ (this reflects a teacher ‘rule’: something can feel different for the other person).
- Teacher: ‘But if you recognize this and notice that Nynke feels bad about it, why don’t you quit and tell the others: “Guys let’s not do this”?’
- Steven: ‘I know why. They [classmates] will then attack or bully Coen because he is protecting Nynke. Everyone responds to each other all the time’.
- Teacher: Yes, that is exactly the problem’.

The excerpt shows how the children do seem to have an understanding of how the dynamics work: people are hurt in their constant reactions to each other, and it would be ideal to consider the feelings of other classmates. The way in which Bente claims her innocence by saying she was not on the ‘bully-list’ exemplifies how this classroom struggles with the discrepancies between ideal behavior according to the teacher’s (adult) empathic and social rules and the children’s real behavior according to their own empathic and social rules on the peer level. The discrepancy or struggle between knowing and doing in social interactions is accurately summarized by the teacher’s remark after a bully incident: ‘You either do not remember our social rules or you refuse to behave accordingly’. In Cl.3, the children demonstrated an understanding of what the teacher wanted and regularly complied with the teacher’s ‘social wishes’; however, they also followed their own rules. In some cases, the classroom and teacher’s rules matched; for example, when someone was bullied or yelled at, it was quite common for another child to intervene, with remarks such as: ‘Stop it, that is not funny, stop!’ However, in other cases, the classroom and teacher’s rules did not match; for example, the peer-to-peer rule to ‘Not cry’ was opposed to the teacher’s encouragement to share sad feelings. The mismatch between teacher and classroom rules and ideas about what was ‘normal’ seemed related to either the difference in cultural background between the children (mainly from Moroccan-Dutch and Turkish-Dutch descent, combined with a Muslim background) and the teachers (both from a Dutch background) and the difference in school-rules and street based peer-rules (El Hadioui, 2011; El Hadioui et al., 2019).

#### The negative versus the positive

While in Cl.2, a common remark would be: ‘Nice jacket, new one?’ (out in the open and positive), in Cl.3, a clearly sarcastic tone was apparent, for example, ‘Niceee shoes, not’ (out in the open and negative), while in Cl.1, a handwritten note about someone in the outgroup who was wearing weird socks (covert and negative) would be typical. The positive vs. negative norm influenced empathic expressions and inferences toward the feelings of others. In Cl.1 it was seldomly seen that one would overtly show vulnerability or respond to a classmate that was in pain or sad for example, whereas in Cl.2 this was much more ‘normal’. In Cl.3, numerous cases of less empathic expressions were observed: from making a harsh remark followed by a ‘do not cry’ to loudly laughing at someone who makes a mistake in a calculation or a sentence in front of the class. However, this way of openly being negative toward each other, without much consideration for someone’s feelings, seemed ‘normal’ in Cl.3: the children were used to this type of communication and often did not seem to be affected by these types of remarks. The overt expressions to each other, which could also be positive in certain situations, marked the way the class interacted. In Cl.2, the pattern of positive encouragement (even when the ‘other’ did not belong to the ingroup) seemed to stimulate prosocial behaviors among the class.

### Observations 3. Individual practices: classroom cases

In every classroom there were individual children who stood out in the observations in relation to empathic expressions, classroom dynamics and/or prosocial behaviors. Here, we describe the children who stood out for being isolated or acting prosocial. Later we will relate these observed classroom positions to measured cognitive abilities and social network scores.

#### Being isolated

In Cl.1, there were three girls and two boys who match this description. One of the girls (Amy) did have a friend with whom she occasionally shared her feelings, but otherwise it was hard to tell what she was thinking. The other two (Inge and Marianne) were often together and excluded by others. One day Inge was crying – which she did quite regularly – because her friend Marianne was being bullied. In her account of the situation, she blamed Bente and Lisanne, two girls with leadership roles in the classroom. Concerning the boys in this classroom, two of them, Dylan and Finn, found themselves regularly excluded from interactive processes within the classroom. Dylan did not always seem very aware of this, but Finn did. The two boys cried relatively often and regularly called in sick. One day when Dylan did not appear at school, it was suggested by the other children that ‘He is not really sick, but does not want to come to school’. In Cl.2, a girl, Hanne, and a boy, Lars, were also excluded from social classroom processes to some extent. Hanne often interacted with the researcher rather than with other children and Lars often played alone during school breaks. Both occasionally made remarks in classroom discussions that showed a lack of sensitivity, either in being too loud, not topic related or already mentioned by someone else. Additionally, one of the girls, Maya, exhibited behavior that was slightly similar to Amy from Cl.1 – she did not appear to express her feelings toward others very often and only shared some observable thoughts and feelings with one or two friends. In Cl.3, two girls, Özge and Nazmiye, and one boy, Asaf, were often excluded – or excluded themselves – from social processes. Another boy, Hamid, did not always appear to understand the social interactions that were going on. Similar to Hanne (Cl.2) and Marianne (Cl.1), Özge showed a preference for interacting with the researcher rather than interacting with her classmates. This was not well received by the other children. Both Özge and Nazmiye decided to not participate in the class summer camp, despite the teacher’s attempts to include them.

#### Acting prosocial

In two cases, the children who had an exemplary role in social interactions also – in their own way – showed prosocial behavior. Marouan, a boy in Cl.3, tended to support children who were not as articulate or who were yelled at, while Lieke, one of the girls in Cl.2, was often involved in helping other children, showing her own emotions, verbally and non-verbally responding to other children’s feelings and emotions, and reflecting on her own actions in terms of whether these were nice to other children or not (and then apologizing). Additionally, in Classrooms 1 and 3 there were two girls (Zoë and Marieke in Cl.1, Alisa and Chaimae in Cl.3) and one boy (Daniel in Cl.1, Marouan in Cl.3) who helped, comforted, mediated and/or included other children despite the general tendency of the classroom to, respectively, exclude (Cl.1) or overshadow (Cl.3) other children on a regular basis. Zoë, for example, was observed helping a classmate who was sad because a mathematics exercise was not going well. Daniel mediated during a group reading exercise that almost degenerated into a fight and told the other three boys to ‘Leave it’ and ‘Let the other person go’, while he put the books back in their original place. Interestingly, Lisanne and Bente (see above) did show sensitivity toward exposed feelings from girls within their ingroup and also actively helped and comforted their ingroup peers; however, outside the ingroup, this prosocial behavior was observed far less often.

### Individual abilities: numbers and observations

#### Classroom distribution

Children’s individual abilities were analysed and mapped by computing z-scores for the three classrooms as a whole (see Methods – Data Analytical Approach). The distribution of these z-scores, indicated a minority of high and low scoring^3^ children were identified in every classroom. This means that generally the distribution of ability scores is not strikingly different between the classrooms.

#### Individual high- and low-scorers

Based on the computed z-scores we subsequently computed and found low and high scorers in our overall sample. To integrate the ability scores and observational findings on an individual level, we examined whether children who had relatively high and low individual ability scores also stood out in the observational findings. It became apparent that there were both matches and mismatches between individual high and low scorers and their more or less empathic behaviors as observed within the classroom.

From the children who were described as ‘being isolated’, the majority had a relatively low score. However, there were also children who were not observed as ‘being isolated’ but still had a low score. Quirine (Cl.1), for example, was one of the low scorers who was regularly mentioned in social interactions, she had friends who stood up for her and she also exhibited empathic insights in group situations; for example, she allowed another child to tag her when she realized the child had been a catcher for a long time. What characterized Quirine was her imperturbability: she would go her own way and did not seem to be overly distracted by what other’s thought about her, which might be partially explained by her relatively weak empathic ability score. Interestingly, there were also children who were observed and described as ‘being isolated’, but who did not have low ability scores. The fact that Özge (Cl.3) and Marianne (Cl.1) did not belong to the low scorers, while their ‘isolated partners’, Nazmiye (Cl.3) and Inge (Cl.1) did, suggests that low ability does not always co-occur with social isolation. Another intriguing finding is that the observed reclusiveness of Amy in Cl.1 seemed unrelated to a relatively low ability, while a similar observed reclusiveness of Maya in Cl.2 did correspond with a relatively low ability score. We found that 6 out of 10 high scorers were observed and described as ‘being prosocial’. Again, the integrated findings revealed a nuanced picture about what it means to be a high scorer. In Cl.1, for example, the four high scorers can clearly not all be put into the same category. While Daniel’s and Zoë’s prosocial behavior and Lisanne’s ambivalent mix of understanding and helping her ingroup peers and manipulating and bullying her outgroup peers was already evident in the observational findings (see above), Floor’s behavior was much less apparent. Floor’s observed behaviors could be characterized as friendly but calm, not that expressive and going with the flow. In the rough data, there are descriptions of her being ‘helpful’ and ‘chatty’, but this was far less noticeable than in the cases of Zoë and Daniel. A similar point can be made about Benno in Cl.2, who was calm, friendly and helpful when needed, but in no way remarkably present in empathic interactions or prosocial actions. Interestingly, there were children in Classrooms 1 and 2 whose prosocial/helping/expressive empathy behavior was far more visible than Floor’s and Benno’s, but whose ability scores were not as high. Clearly, there is not a single type of ‘high scorer’ which was also found to apply to Cl.3.

#### Individual abilities and community abilities

Interestingly, in Cl.1, some children with average to good ability scores were excluded from the social interactions and empathic processes in the classroom, while in Cl.2, only a few children who had low ability scores were sometimes socially excluded. Moreover, in some cases, an individual’s ability score was relatively high or low but this did not directly relate to more or less empathic behaviors, respectively, as observed in the classroom. This relation between being a low or high scorer and standing out in the observations (or vice versa) was much higher in Classrooms 1 and 3 compared to Cl.2. In Cl.1, 75% of low scorers and 75% of high scorers were also observed as either ‘isolated’ or ‘prosocial’; in Cl.2, 60% of the low scorers and 0% of the high scorers were mentioned as ‘isolated’ or ‘prosocial’, respectively; and in Cl.3, 100% of the low scorers and 60% of the high scorers were mentioned. A possible explanation for the unique position of Cl.2 can be that the strong community ability to take care of each other and be positive toward one another makes prosocial behavior the norm. Children who act prosocial do not stand out, since it is a community ability. The community ability overrules the individual ability except for the ‘outliers’ whose individual ability developmentally differ from their peers in the same age group thus. In this type of classroom children’s individual abilities matter less, since the driving force behind expressions of empathy is the community ability.

### Social network analysis: numbers and observations

In the social network analysis (Table 1), we compared the classrooms to each other and obtained some valuable information about how to perceive the contexts of the various classrooms.

**Table 1:** Out = Outdegree (like/dislike/ask for help).

	Cl.1 (*N* = 28)			Cl.2 (*N* = 27)			Cl.3 (*N* = 22)			ANOVA			Mean	SD	Range	Mean	SD	Range	Mean	SD	Range	F (df1, df2)	*p*	*η*^2^
Likes Out	7.18^*1–3^	2.37	6.26–8.10	8.44	3.22	7.17–9.27	9.82^*1–3^	4.73	7.72–11.91	3.57 (2, 74)	0.03	0.09
Likes In	7.18^*1–3^	3.20	5.94–8.42	8.44	2.86	7.31–9.58	9.27^*1–3^	2.88	8.00–10.55	3.31 (2, 74)	0.05	0.08
Dislikes Out	6.79^**1–2, 1–3^	4.19	5.16–8.41	2.89^**1–2^	3.02	1.70–4.08	2.05^**1–3^	2.61	0.89–3.02	14.48 (2, 74)	<0.01	0.28
Dislikes In	6.79^**1–2, 1–3^	3.05	5.60–7.97	2.89^**1–2^	2.59	1.86–3.91	1.95^**1–3^	1.70	1.20–2.71	26.06 (2, 74)	<0.01	0.41
Help Out	4.79^*1–2^	2.81	3.70–5.87	7.89^*1–2^	5.87	5.57–10.21	9.45	5.26	7.12–11.79	6.31 (2, 74)	0.03	0.15
Help In	4.79^**1–2, *1–3^	3.00	3.62–5.95	7.89^**1–2^	3.40	6.54–9.23	9.09^*1–3^	3.62	7.48–10.70	11.47 (2, 74)	<0.01	0.24

In = Indegree (being disliked/liked/asked for help) for Classroom 1 (Cl.1), Classroom 2 (Cl.2), and Classroom 3 (Cl.3) *post hoc* (Bonferroni). *p < 0.05, **p < 0.01. 1–2 pairwise comparison Cl1–Cl2, 1–3 pairwise comparison CL1–Cl3.

The significant differences between the classrooms were striking particularly in the dislike- and the help-network Moreover, the effect sizes turned out to be medium-large which confirms a relevant (besides the already presented significance) difference in social network composition between the classrooms. Cl.1 stands out in comparison to the other two classrooms: there were relatively more dislikes, and relatively fewer reported help requests, which means that the frequency of asking and being asked for help is relatively low in Cl.1. Moreover, the mean score for dislikes (indegree and outdegree) in Cl.1 was more than twice as high as in Classrooms 2 and 3. In Cl.1, this could be clearly linked to the observed *ingroup and outgroup* dynamics, in combination with a focus on the *negative*. Interestingly, the focus on the negative was also present in Cl.3; however, in this case, it did not seem to be directly related to disliking others. A possible reason for this is that the behavior of the children observed by the researcher was experienced and understood differently by the children themselves. Children in Cl.3 may have been used to blunt remarks such as: ‘Shut up you cow!’, or perceived these as regular classroom dynamics that did not directly imply deeper relational problems. Interestingly, the importance of ingroup vs. outgroup processes in Cl.1 generated a context in which *whom* to ask was more important than *what* to ask. Additionally, we see in Classrooms 1 and 2 that children who were asked for help were all part of the ‘plusklas’, which means they participated in alternative lessons to remain cognitively stimulated. Who the children would choose to go to for help thus seemed related to two observable factors: a willingness to ask another child based on the relationship; and the perceived cognitive ability of the other child to help with school work. Motivations relating to whom to go to and for what reason thus also clearly differed in each classroom. In summary, part of the social network results can be directly related to the shared empathic practices and norms as observed in every classroom. However, whereas in the observations Cl.2 stood out as compared to Cl.1 and 3, with Cl.2 showing much more overt and positive empathic practices on group level, here it is Cl.1 that stands out showing significantly more dislikes and less willingness to help. Cl.3 thus, seems to have indeed two different faces (see earlier) which we will come back at in the Discussion section below.

## Discussion

The main purpose of this research was to explore how the combination of the theoretical frameworks and methodological tools of two disciplines – anthropology and psychology – can provide new insights on children’s empathy. The key findings of our study came from the integration of the three levels of investigation – observational data, psychological tasks and social network data. Main research questions (RQ) were:

1. How is the child’s individual ability to empathize related to their empathic expressions as observed in the socio-cultural environment of their daily school lives?
2. How can the use of social network analysis help us to find the interplay between empathy as an individual construct and empathy as part of a classroom community of practice?

Below we answer these research questions in three detailed subsections. It should be kept in mind that our primary aim at this point is not to provide generalizable insights on the construct of empathy in children, but to explore how our multidisciplinary approach can be applied within this topic and what kind of new insights this can potentially generate.

### RQ 1: rule based prosocial behaviors or empathy based prosocial behaviors

In our findings there was a discrepancy between the roughly similar distribution of individual scores between the classrooms and the very different empathic behaviors and classroom rules. Bethlehem et al. (2017) pose that empathy is only one of the two possible motivators of helping others and related prosocial behaviors. The other motivator is a rule based/system based morality (*ibid*: p 744). Could it be that in the one classroom, or the one case, prosocial behavior is rule-induced (see Cl.1 and their willingness to follow the ‘rules’, but only when the teacher is there) and in the other it is more empathy-induced (Cl 2. in which cooperation seemed an inbuilt behavior)? This might explain part of the discrepancy between empathic ability scores and empathic expressions and prosocial behaviors in our study. Especially in the age group of our study (9–12) it is said that autonomy is still developing even far into adolescence (Wray-Lake et al., 2010), which makes it plausible that rules that belong to the authority of the teacher can play a substantial role in behavior. Another possible explanation for the discrepancy may be that children’s participation in empathic behaviors is based more on community abilities than on individual abilities.

### RQ 1: individual empathic abilities and collective empathic abilities

In the integration of the observational findings and the individual ability scores interesting overlap and discrepancy occurred. The general overlap tells us that most children who had a relative low ability score also stood out in the observations as ‘isolated’ in some sort of way and children who were observed as strikingly prosocial had in general more often a relative high ability score. This overlap shows us how individual abilities do matter since they indicate a certain position or type of behavior in the classroom. For social scientists this is important to consider, it can help them to more relevantly direct their attention during observational research and to interpret deviant empathic behavior in terms of processes and abilities. At the same time, marked differences in empathic behaviors between classrooms could not be traced back to between classroom differences in average level of individual abilities. This particular discrepancy that occurred between classrooms (as opposed to the general overlap) tells us an exciting additional story. In Cl.2 children’s relative individual ability score had a markedly smaller effect on the possibility that this child would stand out as either prosocial (high score) or isolated (low score) than in Cl.3 and 1. This finding suggests that the shared practices and the accompanying community norms (Payne et al., 2020) within the classroom – the community of practice - can be highly influential on the empathic expressions and prosocial behaviors that can be observed and even overrule a certain individual ability score composition of a classroom. The community ability partly overrules the individual ability. This means that in each specific classroom children’s individual abilities can matter more or less, since a driving force behind expressions of empathy is the community ability. It is, in the end, the interplay between individual and community abilities that gives the most complete information.

### RQ 2: SNA findings and peer cultures and school culture

The social network analysis (SNA) showed that it was Cl.1 that significantly differed from the other two classrooms in both the dislike- and the help-network. At first sight this was unexpected. It was Cl.2 that was distinct from Cl.1 and Cl.3 in how prosocial behaviors were showed and how community abilities prevailed over individual abilities. It was Cl.3 that was distinct from Cl.1 and Cl.2 as related to SES: the geographical area and its related income level which was below national average in Cl.3 and above average in Cl.1 and Cl.2 and cultural background was not so diverse and mainly Wester in Cl.1 and Cl.2 and super diverse and partly non-Western in Cl.3. So how could it be that in the SNA Cl.1 significantly differed from the other two Classrooms?. Two lines of thought are possible to grasp the meaning of this finding. First, we see in the extensive work of Kyratzis (2004) how she highlights ‘inclusion and power in the peergroup’ (Kyratzis, 2004: 627). Interestingly, Cl 1 did stand out on these important peer culture concerns: inclusion/exclusion processes were relatively dominant and important, there was a lot of gossip talk, teasing and conflict in games and so called ‘weak boys, and girls (…) were derided by the ringleaders’ (*ibid* p.635). In Cl 2 the need to power play did not play such a significant role. In Cl 3 children were not at all short of direct confrontation (*ibid* p.632), however the power play was often seen in relation to the teacher’s role and did not result in a relative high amount of dislikes. This brings us to an interesting second line of thought: the difference between peer culture and school culture. It has been suggested that children either feel motivated to participate in the school culture and climb on the school ladder or they feel more motivated to participate in the peer culture – which is related to street culture – and climb on the peer-ladder (El Hadioui, 2011; El Hadioui et al., 2019). In Cl 3 climbing on the peer-culture/street-culture ladder seemed more important and therefore the conflict was *between* the school-culture and the peer-culture and not *within* the peer culture between various subgroups as in Cl.1. This between/within-difference might be part of our understanding as to why in Cl.3 the willingness to help each other within the peer to peer interaction was much larger than this same willingness in Cl.1.

### Implications, limitations and conclusion

By answering the main research questions we hope to have made a convincing case about the quality and contribution of this interdisciplinary study to the field of children’s empathy and the context of the classroom. However, this research also has limitations. First, there will always be missing information in ‘real-life’ research: the researchers were not in the classroom every day and there will always be events, discussions and interactions that are not observed. Second, the psychological tasks tests in the current research included mainly cognitive empathy tasks whilst the mapped expressions and behaviors include cognitive empathy and affective empathy, concern and emotional contagion. Third, in terms of interpreting and using our quantitative data it would have been better to have a larger group sample. Due to the setup of this research, in which we combined our quantitative dataset with long-term observations which had an essential role in our exploration of the topic, this was not feasible. Fourth, the internal consistency of the RME-Child turned out to be low which has been reported earlier (Van der Meulen et al., 2017: 2). However, because we used a combination of measures to assess ability, we believe this has not had too much impact on the findings. Fifth, we do believe that gender can be an interesting element in future research on empathic abilities – both on individual - and community level. In our study it was beyond the scope of this article and unfortunately the group was too small. Sixth, we did not include linguistic background of the children which might have been of influence on both the test results and their observed social behaviors. Seventh, our focus on the children and their thoughts meant that we did not directly involve teachers. Interviews with the teachers would form a rich additional source of information in further research. Last, but not less important: in combining qualitative and quantitative measures we had to do justice to the different purposes and criteria these methodologies can have (Tracy, 2010). Because our primary goal is exploring integrated insights in daily processes and including valid and rich qualitative data is essential within this goal, we prioritized sufficient depth and detail in our data over obtaining a large sample. The latter would be more suited and feasible if the aim and set-up are primarily quantitative. Besides limitations, this research also entails some valuable implications. To begin with, this research demonstrates that combining theoretical starting points from psychology, education and anthropology has important added value. It was only in the integration of data, that the main insights were revealed: the possible influence of rule-based prosocial behaviors versus empathy based prosocial behaviors, the interplay between community empathic abilities and individual empathic abilities, and the role of peer culture and school culture. Future research might focus on developing a greater understanding of the interplay of the various factors mentioned. A further aim could be to enhance the practical use of these insights into empathy to improve social skills interventions at schools, (Joronen et al., 2011; Schonert-Reichl et al., 2012). As Ramaswamy and Bergin (2009) already specified a decade ago: ‘interventions could be tailored to specific classrooms’ (…). The idea that a specific classroom has a specific need that is related to a certain dynamic of (pro)social behaviors is directly echoed in our findings. We conclude by encouraging researchers to incorporate multiple research methods within one study and not hesitate to work intensively with researchers in other disciplines. It may not always be easy as Main et al. (2017) already predicted, but it is only by combining the strengths of multiple research fields that we can gain unique and valuable into the how, the when and the why of complex processes such as children’s empathy.

## Data availability statement

The raw data supporting the conclusions of this article will be made available by the authors, without undue reservation.

## Ethics statement

The studies involving human participants were reviewed and approved by Ethical Committee of the Department of Psychology and Education of the VU University. Written informed consent for participation was not provided by the participants’ legal guardians/next of kin because: Written informed consent was provided for 2 of the 3 classrooms. Teachers of the other classroom chose - in accordance with the first author - to work with passive written and oral consent. The main reason for this type of consent was that some of the parents of the children who participated were not able to read the Dutch consent form, since their lingual background is non-Dutch.

## Author contributions

SR developed the concept of the research in collaboration with LK, SE, and FW. The data collection was done primarily by SR. SR and FW analyzed the data, supervized by LK. SR, FW, SE, and LK interpreted the results. SR wrote the manuscript guided by LK and FW who also contributed. AM, LK, FW, and SE provided critical comments on the manuscript at several stages. AM and LK provided comments at the last stage of the manuscript. All authors contributed to the article and approved the submitted version.

## Funding

LK was funded by a VICI grant from the Netherlands Organization for Scientific Research. Grant number 453-11-005.

## Conflict of interest

The authors declare that the research was conducted in the absence of any commercial or financial relationships that could be construed as a potential conflict of interest.

## Publisher’s note

All claims expressed in this article are solely those of the authors and do not necessarily represent those of their affiliated organizations, or those of the publisher, the editors and the reviewers. Any product that may be evaluated in this article, or claim that may be made by its manufacturer, is not guaranteed or endorsed by the publisher.

## Footnotes

^1^*All of the names of the children have been changed to protect the privacy of our participants.

^2^In the current paper, ‘ingroups’ means: the peers who are considered to be friends; ‘outgroups’ means: the peers who are considered to be everybody else.

^3^The low or high scorers were individuals whose standard deviations were below or above 1 on multiple tasks, respectively. The tasks used were: RME, LEAS-C, HIFDS.

## References

[ref1] Ahn R. P. Goh D. H. (2010). Cyberbullying among adolescents: the role of affective and cognitive empathy, and gender. Child Psychiatry Hum. Dev. 41, 387–397. doi: 10.1007/s10578-010-0176-3 20238160

[ref2] Astuti R. (2007). Weaving together culture and cognition: an illustration from Madagascar. Intellectica Revue de l'Association Pour la Recherche Cognitive 46, 173–189.

[ref3] Astuti R. (2015). Implicit and explicit theory of mind. Anthropol Century. 13, 636–650.

[ref4] Bajgar J. Ciarrochi J. Lane R. Deane F. P. (2005). Development of the levels of emotional awareness scale for children (LEAS-C). Br. J. Dev. Psychol. 23, 569–586. doi: 10.1348/026151005X35417, PMID: 21214598

[ref5] Banerjee R. Watling D. Caputi M. (2011). Peer relations and the understanding of faux pas: longitudinal evidence for bidirectional associations. Child Dev. 82, 1887–1905. doi: 10.1111/j.1467-8624.2011.01669.x, PMID: 22023260

[ref6] Baron-Cohen S. Wheelwright S. Spong A. Scahill V. Lawson J. (2001). Studies of theory of mind: are intuitive physics and intuitive psychology independent? J. Dev. Learn. Disord. 5, 47–78.

[ref7] Batson C. D. (2009). “These things called empathy: Eight related but distinct phenomena” in The social neuroscience of empathy (pp. 3–15). Boston Review. eds. Decety J. Ickes W. doi: 10.7551/mitpress/9780262012973.003.0002

[ref8] Benedict R. (1938) in General Anthropology. ed. Boas F. (Boston: DC Heath), 1938.

[ref9] Bethlehem R. A. Allison C. van Andel E. M. Coles A. I. Neil K. Baron-Cohen S. (2017). Does empathy predict altruism in the wild? Soc. Neurosci. 12, 743–750. doi: 10.1080/17470919.2016.1249944, PMID: 27759496

[ref10] Blair R. J. R. (2005). Responding to the emotions of others: dissociating forms of empathy through the study of typical and psychiatric populations. Conscious Cogn 14, 698–718. doi: 10.1016/j.concog.2005.06.004 16157488

[ref11] Blair R. J. R. (2018). Traits of empathy and anger: implications for psychopathy and other disorders associated with aggression. Philos Trans R Soc Lond B Biol Sci 373:155. doi: 10.1098/rstb.2017.0155 PMC5832681 29483341

[ref001] Borgatti S. P. Mehra A. Brass D. J. Labianca G. (2009). Network analysis in the social sciences. science 323, 892–895. 19213908 10.1126/science.1165821

[ref12] Borgatti S. P. (2002). NetDraw: Graph Visualization Software. Harvard: Analytic Technologies.

[ref13] Borgatti S. P. Everett M. G. Freeman L. C. (2002). UCINET for Windows: Software for Social Network Analysis. Harvard: Analytic Technologies.

[ref14] Briggs J. L. (2008). Daughter and pawn: one ethnographer’s routes to understanding children. Ethos 36, 449–456. doi: 10.1111/j.1548-1352.2008.00026.x

[ref15] Caravita S. Di Blasio P. Salmivalli C. (2009). Unique and interactive effects of empathy and social status on involvement in bullying. Soc. Dev. 18, 140–163. doi: 10.1111/j.1467-9507.2008.00465.x

[ref16] Castro V. L. Halberstadt A. G. Garrett-Peters P. (2016). A three-factor structure of emotion understanding in third-grade children. Soc. Dev. 25, 602–622. doi: 10.1111/sode.12162, PMID: 29129962 PMC5679467

[ref17] Coll M.-P. Viding E. Rütgen M. Silani G. Lamm C. Catmur C.. (2017). Are we really measuring empathy? Proposal for a new measurement framework. Neurosci Biobehav Rev 83, 132–139. doi: 10.1016/j.neubiorev.2017.10.009 29032087

[ref18] Corbin J. Strauss A. (2008). Basics of Qualitative Research: Techniques and Procedures for Developing Grounded Theory (3rd ed.). Available at: https://paloaltou.idm.oclc.org/login?url=http://search.ebscohost.com/login.aspx?direct=true&db=psyh&AN=2008–05815-000 (Retrieved spring 2022).

[ref19] Creswell J. W. Plano Clark V. L. Gutmann M. L. Hanson W. E. (2003). “Advanced mixed methods research designs” in Handbook of Mixed Methods in Social and Behavioral Research eds. Tashakkori A. Teddlie C. (Thousand Oaks, CA: Sage Publications), 209–240.

[ref20] Cuff B. M. P. Brown S. J. Taylor L. Howat D. J. (2016). Empathy: a review of the concept. Emot. Rev. 8, 144–153. doi: 10.1177/1754073914558466

[ref21] Davis M. H. (1983). Measuring individual differences in empathy: evidence for a multidimensional approach. J. Pers. Soc. Psychol. 44, 113–126. doi: 10.1037/0022-3514.44.1.113

[ref22] Decety J. (2010). The neurodevelopment of empathy in humans. Dev. Neurosci. 32, 257–267. doi: 10.1159/000317771, PMID: 20805682 PMC3021497

[ref23] Decety J. Jackson P. L. (2004). The functional architecture of human empathy. Behav. Cogn Neurosci Rev 3, 71–100. doi: 10.1177/1534582304267187 15537986

[ref24] Decety J. Lamm C. (2006). Human empathy through the lens of social neuroscience. Sci. World J. 6, 1146–1163. doi: 10.1100/tsw.2006.221, PMID: 16998603 PMC5917291

[ref25] Demetriou H. (2018). “A study of empathy in the early and middle childhood years” in Empathy, Emotion and Education (London: Palgrave Macmillan).

[ref26] Eisenberg N. (2000). Emotion, regulation, and moral development. Annu. Rev. Psychol. 51, 665–697. doi: 10.1146/annurev.psych.51.1.665 10751984

[ref27] El Hadioui I. (2011). Hoe de Straat de School Binnendringt. Denken Vanuit de Pedagogische Driehoek van de Thuiscultuur, de Schoolcultuur en de Straatcultuur. Amsterdam: Van Gennep.

[ref28] El Hadioui I. Slootman M. El-Akabawi Z. Hammond M. Mudde A. L. Schouwenburg S. (2019). Switchen en klimmen: Over het switchgedrag van leerlingen en de klim op de schoolladder in een grootstedelijke omgeving. Van Gennep.

[ref29] Engelen E.-M. Röttger-Rössler B. (2012). Current disciplinary and interdisciplinary debates on empathy. Emot. Rev. 4, 3–8. doi: 10.1177/1754073911422287

[ref002] Fotopoulou E. Zafeiropoulos A. Alegre A. (2019). Improving social cohesion in educational environments based on a sociometric-oriented emotional intervention approach. Education Sciences 9:24.

[ref003] Fotopoulou E. Zafeiropoulos A. Papavassiliou S. (2021a). EmoSocio: An open access sociometry-enriched Emotional Intelligence model. Current Research in Behavioral Sciences 2:100015

[ref004] Fotopoulou E. Zafeiropoulos A. Guiu I. M. Feidakis M. Daradoumis T. Papavassiliou S. (2021b). “Assessing students’ social and emotional competencies through graph analysis of emotional-enriched sociograms” in Intelligent Systems and Learning Data Analytics in Online Education (Academic Press), 239–271.

[ref30] Gilin D. Maddux W. W. Carpenter J. Galinsky A. D. (2013). When to use your head and when to use your heart: the differential value of perspective-taking versus empathy in competitive interactions. Personal. Soc. Psychol. Bull. 39, 3–16. doi: 10.1177/0146167212465320 23150199

[ref31] Groark K. P. (2008). Social opacity and the dynamics of empathic in-sight among the Tzotzil Maya of Chiapas, Mexico. Ethos 36, 427–448. doi: 10.1111/j.15481352.2008.00025.x

[ref32] Gutiérrez K. D. Rogoff B. (2003). Cultural ways of learning: individual traits or repertoires of practice. Educ. Res. 32, 19–25. doi: 10.3102/0013189X032005019

[ref33] Haneda M. (2006). Classrooms as communities of practice: a reevaluation. TESOL Q. 40, 807–817. doi: 10.2307/40264309

[ref34] Harris P. L. (1994). The child's understanding of emotion: developmental change and the family environment. J. Child Psychol. Psychiatry 35, 3–28. doi: 10.1111/j.1469-7610.1994.tb01131.x 8163629

[ref35] Hay D. F. Payne A. Chadwick A. (2004). Peer relations in childhood. J. Child Psychol. Psychiatry 45, 84–108. doi: 10.1046/j.0021-9630.2003.00308.x 14959804

[ref005] Hollan D. (2012). Emerging issues in the cross-cultural study of empathy. Emotion Review 4, 70–78.

[ref36] Hollan D. Throop J. C. (2008). Whatever happened to empathy? introduction. Ethos 36, 385–401. doi: 10.1111/j.1548-1352.2008.00023.x

[ref37] Hollan D. Throop J. C. (Eds.). (2011). The Anthropology of Empathy: Experiencing the Lives of Others in Pacific Societies. Berghahn Books. Available at: http://books.google.com/books?hl=en&lr=&id=CGCCcGVcrVcC&pgis=1 (Retrieved spring 2022).

[ref38] Johnson R. B. Onwuegbuzie A. Turner L. A. (2007). Toward a definition of mixed methods research. J. Mixed Methods Res. 1, 112–133. doi: 10.1177/1558689806298224

[ref39] Jordan M. R. Amir D. Bloom P. (2016). Are empathy and concern psychologically distinct? Emotion 16, 1107–1116. doi: 10.1037/emo0000228, PMID: 27668802

[ref40] Joronen K. Häkämies A. Åstedt-Kurki P. (2011). Children’s experiences of a drama programme in social and emotional learning. Scand. J. Caring Sci. 25, 671–678. doi: 10.1111/j.1471-6712.2011.00877.x, PMID: 21362006

[ref41] Keysers C. Gazzola V. (2014). Dissociating the ability and propensity for empathy. Trends Cogn. Sci. 18, 163–166. doi: 10.1016/j.tics.2013.12.011, PMID: 24484764 PMC4560165

[ref42] Kyratzis A. (2004). Talk and interaction among children and the co-construction of peer groups and peer culture. Annu. Rev. Anthropol. 33, 625–649. doi: 10.1146/annurev.anthro.33.070203.144008

[ref006] Lave J. Wenger E. (1991). Situated learning: Legitimate peripheral participation Cambridge university press.

[ref43] Lave J. Wenger E. (1999). Legitimate Peripheral Participation. Learners, Learning and Assessment, London: The Open University, 83–89.

[ref44] Leung B. P. Silberling J. (2006). Using Sociograms to identify social status in the classroom. California Sch Psychol 11, 57–61. doi: 10.1007/BF03341115

[ref45] Lieberman M. D. (2007). Social cognitive neuroscience: a review of core processes. Annu. Rev. Psychol. 58, 259–289. doi: 10.1146/annurev.psych.58.110405.085654 17002553

[ref007] Main A. Walle E. A. Kho C. Halpern J. (2017). The interpersonal functions of empathy: A relational perspective. Emotion Review 9, 358–366.

[ref46] Marin A. Wellman B. (2011). “Social network analysis: an introduction” in The SAGE Handbook of Social Network Analysis, eds. Scott J. Carrington P.J.. (London: SAGE Publications) 11–25.

[ref47] Marsh A. A. Finger E. C. Fowler K. A. Adalio C. J. Jurkowitz I. T. N. Schechter J. S.. (2013). Empathic responsiveness in amygdala and anterior cingulate cortex in youths with psychopathic traits. J. Child Psychol. Psychiatry 54, 900–910. doi: 10.1111/jcpp.12063, PMID: 23488588 PMC3716835

[ref48] Martín-Antón L. J. Monjas M. I. Garcia Bacete F. J. Jiménez-Lagares I. (2016). Problematic social situations for peer-rejected students in the first year of elementary school. Front. Psychol. 7:1925. doi: 10.3389/fpsyg.2016.01925 28018265 PMC5156692

[ref008] Meyer M. L. Masten C. L. Ma Y. Wang C. Shi Z. Eisenberger N. I.. (2013). Empathy for the social suffering of friends and strangers recruits distinct patterns of brain activation. Social cognitive and affective neuroscience 8, 446–454. 22355182 10.1093/scan/nss019 PMC3624958

[ref49] Moreno J. L. (1953). Who Shall Survive? Foundations of Sociometry, Group Psychotherapy and Sociodrama; Beacon House: Oxford, England, 8, 173–174.

[ref50] Morse J. M. Niehaus L. (2009). Principles and Procedures of Mixed Methods Design. Walnut Creek, CA: Left Coast Press.

[ref51] Payne K. A. Adair J. K. Colegrove K. S. S. Lee S. Falkner A. McManus M.. (2020). Reconceptualizing civic education for young children: recognizing embodied civic action. Educ Citizenship Soc Justice 15, 35–46. doi: 10.1177/1746197919858359

[ref52] Prevost M. Carrier M. E. Chowne G. Zelkowitz P. Joseph L. Gold I. (2014). The Reading the mind in the eyes test: validation of a French version and exploration of cultural variations in a multi-ethnic city. Cogn. Neuropsychiatry 19, 189–204. doi: 10.1080/13546805.2013.823859, PMID: 23937473

[ref53] Ramaswamy V. Bergin C. (2009). Do reinforcement and induction increase prosocial behavior? Results of a teacher-based intervention in preschools. J. Res. Child. Educ. 23, 527–538. doi: 10.1080/02568540909594679

[ref54] Roepstorff A. Frith C. (2012). Neuroanthropology or simply anthropology? Going experimental as method, as object of study, and as research aesthetic. Anthropol Theory 12, 101–111. doi: 10.1177/1463499612436467

[ref55] Roerig S. Wesel F. Evers S. J. T. M. Krabbendam L. (2015). Researching children's individual empathic abilities in the context of their daily lives: the importance of mixed methods. Front. Neurosci. 9:261. doi: 10.3389/fnins.2015.00261 26283901 PMC4516888

[ref56] Röttger-Rössler B. Scheidecker G. Funk L. Holodynski M. (2015). Learning (by) feeling: A cross-cultural comparison of the socialization and development of emotions. Ethos 43, 187–220. doi: 10.1111/etho.12080

[ref57] Scheler M. (2017). The nature of sympathy Routledge.

[ref58] Schonert-Reichl K. A. Smith V. Zaidman-Zait A. Hertzman C. (2012). Promoting children’s prosocial behaviors in school: impact of the ‘roots of empathy’ program on the social and emotional competence of school-aged children. Sch. Ment. Heal. 4, 1–21. doi: 10.1007/s12310-011-9064-7

[ref59] Singer T. (2006). The neuronal basis and ontogeny of empathy and mind reading: review of literature and implications for future research. Neurosci. Biobehav. Rev. 30, 855–863. doi: 10.1016/j.neubiorev.2006.06.011, PMID: 16904182

[ref60] Sobieski C. Dell'Angelo T. (2016). Sociograms as a tool for teaching and learning: discoveries from a teacher research study. Educ. Forum 80, 417–429. doi: 10.1080/00131725.2016.1207734

[ref61] Throop C. J. (2010). Latitudes of loss: on the vicissitudes of empathy. Am. Ethnol. 37, 771–782. doi: 10.1111/j.1548-1425.2010.01284.x

[ref62] Tracy S. J. (2010). Qualitative quality: eight “big-tent” criteria for excellent qualitative research. Qual. Inq. 16, 837–851. doi: 10.1177/1077800410383121

[ref63] Van der Meulen A. Roerig S. de Ruyter D. van Lier P. Krabbendam L. (2017). A comparison of children's ability to read children's and adults' mental states in an adaptation of the Reading the mind in the eyes task. Front. Psychol. 8:594. doi: 10.3389/fpsyg.2017.00594, PMID: 28491043 PMC5405343

[ref64] Van Doesum N. J. Van Lange D. A. W. Van Lange P. A. M. (2013). Social mindfulness: skill and will to navigate the social world. J. Pers. Soc. Psychol. 105, 86–103. doi: 10.1037/a0032540, PMID: 23647176

[ref65] Vasileiou K. Barnett J. Thorpe S. Young T. (2018). Characterising and justifying sample size sufficiency in interview-based studies: systematic analysis of qualitative health research over a 15-year period. BMC Med. Res. Methodol. 18, 1–18. doi: 10.1186/s12874-018-0594-7 30463515 PMC6249736

[ref66] Warrier V. Grasby K. L. Uzefovsky F. Toro R. Smith P. Chakrabarti B.. (2018). Genome-wide meta-analysis of cognitive empathy: heritability, and correlates with sex, neuropsychiatric conditions and cognition. Mol. Psychiatry 23, 1402–1409. doi: 10.1038/mp.2017.122, PMID: 28584286 PMC5656177

[ref67] Wasserman S. Faust K. (1994). Social network analysis: Methods and applications Cambridge University Press.

[ref68] Whitehead C. (2012). Why the behavioral sciences need the concept of the culture-ready brain. Anthropol Theory 12, 43–71. doi: 10.1177/1463499612436464

[ref69] Winczewski L. A. Bowen J. D. Collins N. L. (2016). Is empathic accuracy enough to facilitate responsive behavior in dyadic interaction? Distinguishing ability from motivation. Psychol Sci 27, 394–404. doi: 10.1177/0956797615624491, PMID: 26847609

[ref70] Wölfer R. Cortina K. S. Baumert J. (2012). Embeddedness and empathy: how the social network shapes adolescents’ social understanding. J. Adolesc. 35, 1295–1305. doi: 10.1016/j.adolescence.2012.04.015, PMID: 22681757

[ref71] Wray-Lake L. Crouter A. C. McHale S. M. (2010). Developmental patterns in decision-making autonomy across middle childhood and adolescence: European American parents’ perspectives. Child Dev. 81, 636–651. doi: 10.1111/j.1467-8624.2009.01420.x, PMID: 20438465 PMC2864944

[ref72] Yang C.-Y. Decety J. Lee S. Chen C. Cheng Y. (2009). Gender differences in the mu rhythm during empathy for pain: an electroencephalographic study. Brain Res. 1251, 176–184. doi: 10.1016/j.brainres.2008.11.062, PMID: 19083993

[ref73] Zahavi D. (2010). Empathy, embodiment and interpersonal understanding: from Lipps to Schutz. Inquiry 53, 285–306. doi: 10.1080/00201741003784663

[ref74] Zaki J. Ochsner K. N. (2012). The neuroscience of empathy: progress, pitfalls and promise. Nat. Neurosci. 15, 675–680. doi: 10.1038/nn.3085, PMID: 22504346

